# METABOLIC SYNDROME, LIFESTYLE FACTORS, AND SLEEP-DISORDERED BREATHING IN US HISPANICS: IMPLICATIONS FOR MID LIFE

**DOI:** 10.1093/geroni/igad104.2442

**Published:** 2023-12-21

**Authors:** Shannon Richard, Du Feng, Dieu-My Tran, Brenna Renn, Jinyoung Kim

**Affiliations:** University of Nevada, Las Vegas, Las Vegas, Nevada, United States; University of Nevada, Las Vegas, Las Vegas, Nevada, United States; University of Nevada, Las Vegas, Las Vegas, Nevada, United States; University of Nevada, Las Vegas, Las Vegas, Nevada, United States; University of Nevada, Las Vegas, Las Vegas, Nevada, United States

## Abstract

US Hispanics/Latinos are disproportionately susceptible to metabolic syndrome (MetS), attributed in part to health and lifestyle factors such as low physical activity levels, diet quality, alcohol use, tobacco use, and sleep-disordered breathing. The risk of MetS and MetS-related complications increases with age. We examined the relationships between select health and lifestyle factors and MetS among Hispanic gender-heritage subgroups and determined whether gender and heritage moderate those relationships. Participants included 14,155 Hispanic Americans aged 18–76 (59% women, mean age 45.92 ± 13.97) from seven heritage subgroups. This secondary analysis of cross-sectional data from the observational Hispanic Community Health Study / Study of Latinos dataset used multinomial logistic regression and Hayes’ PROCESS macro to test these relationships; the dependent variable, MetS, included four categories delineating MetS and related medication use. Results include low physical activity (p< .001) and sleep-disordered breathing (p< .001) were associated with the MetS with medication use group. High alcohol use decreased the probability of being in this group (p< .001). Cigarette pack years were not significantly associated with MetS outcomes. Gender moderated the association between MetS and diet quality (p< .001), alcohol use (p< .001), cigarette pack-years (p< .001), and sleep-disordered breathing (p< .001). Low physical activity and sleep-disordered breathing was significantly associated with MetS group membership, whereas low and high alcohol use were associated with decreased MetS risk. Gender-heritage differences were prominent in each of the study variables. Midlife may be an important target for prevention and intervention; other considerations for aging will be discussed.

